# Supplementary figures and images for: Ubiquitination of stalled ribosomes enables mRNA decay via HBS-1 and NONU-1 in vivo
Source: PLoS Genet. 2023 Jan 10;19(1):e1010577. doi: 10.1371/journal.pgen.1010577 (PMC9870110; doi:10.1371/journal.pgen.1010577)

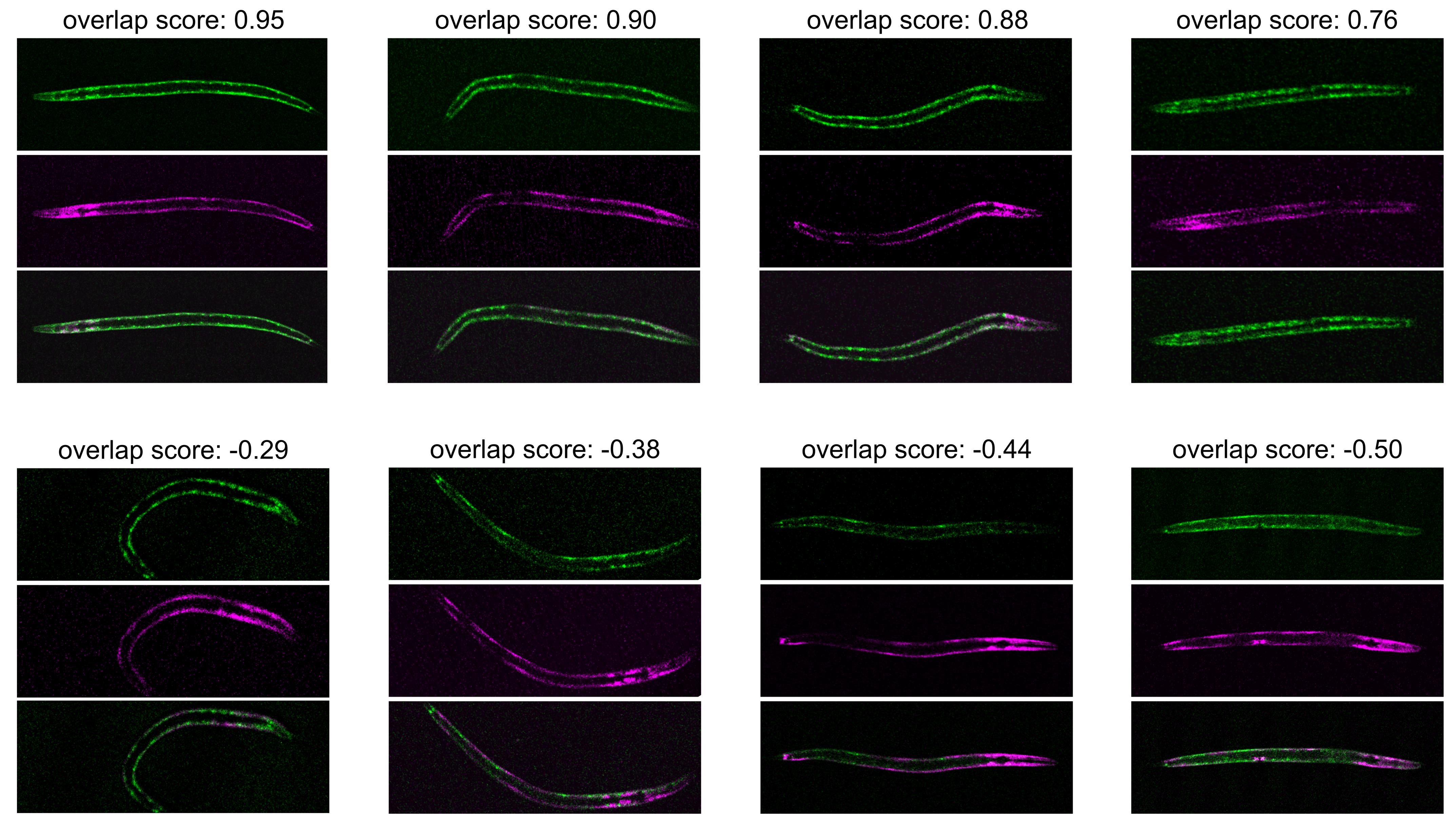

Supplement: S1 Fig — GFP and mCherry images of representative animals expressing unc-54(rareArg) and an mCherry-tagged array. Above is the calculated overlap score. (TIF) [file pgen.1010577.s003.tif]

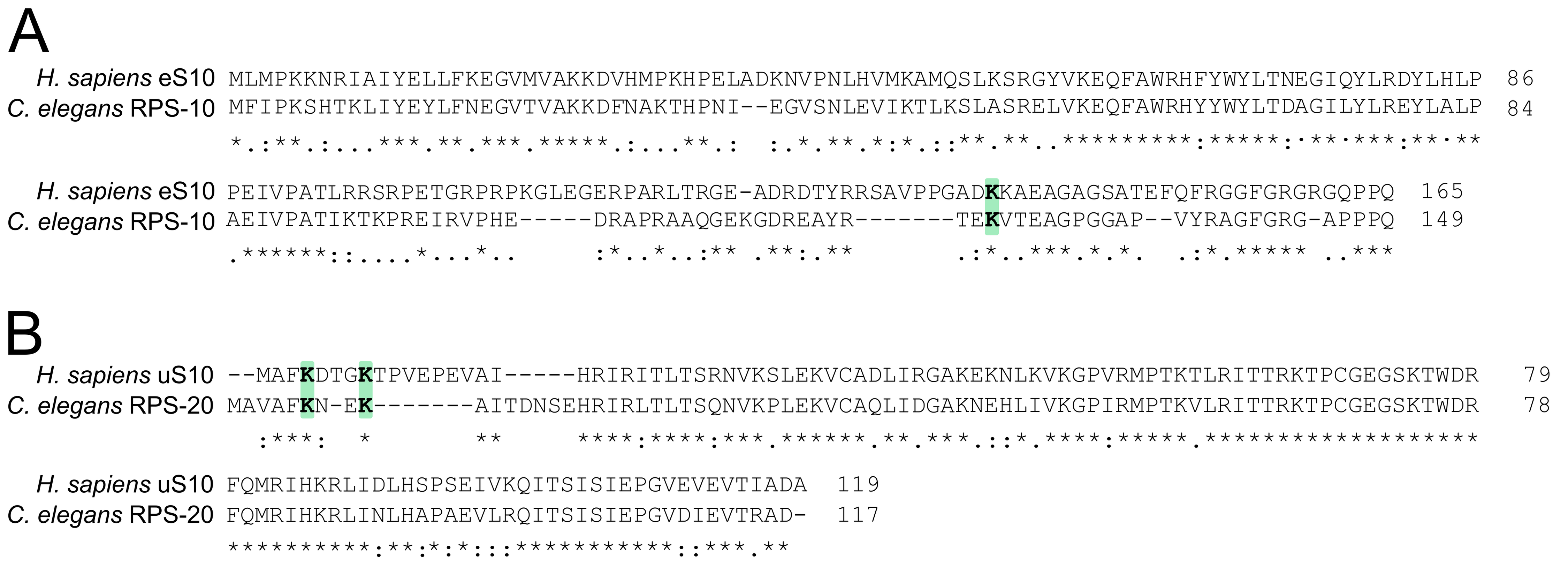

Supplement: S2 Fig — (A) Pairwise sequence alignment of H. sapiens eS10 and C. elegans RPS-10. K125 is highlighted in green. Conservation is as shown in Fig 3A. (B) As in (A), showing H. sapiens uS10 and C. elegans RPS-20 with K6 and K9 highlighted in green. (TIF) [file pgen.1010577.s004.tif]

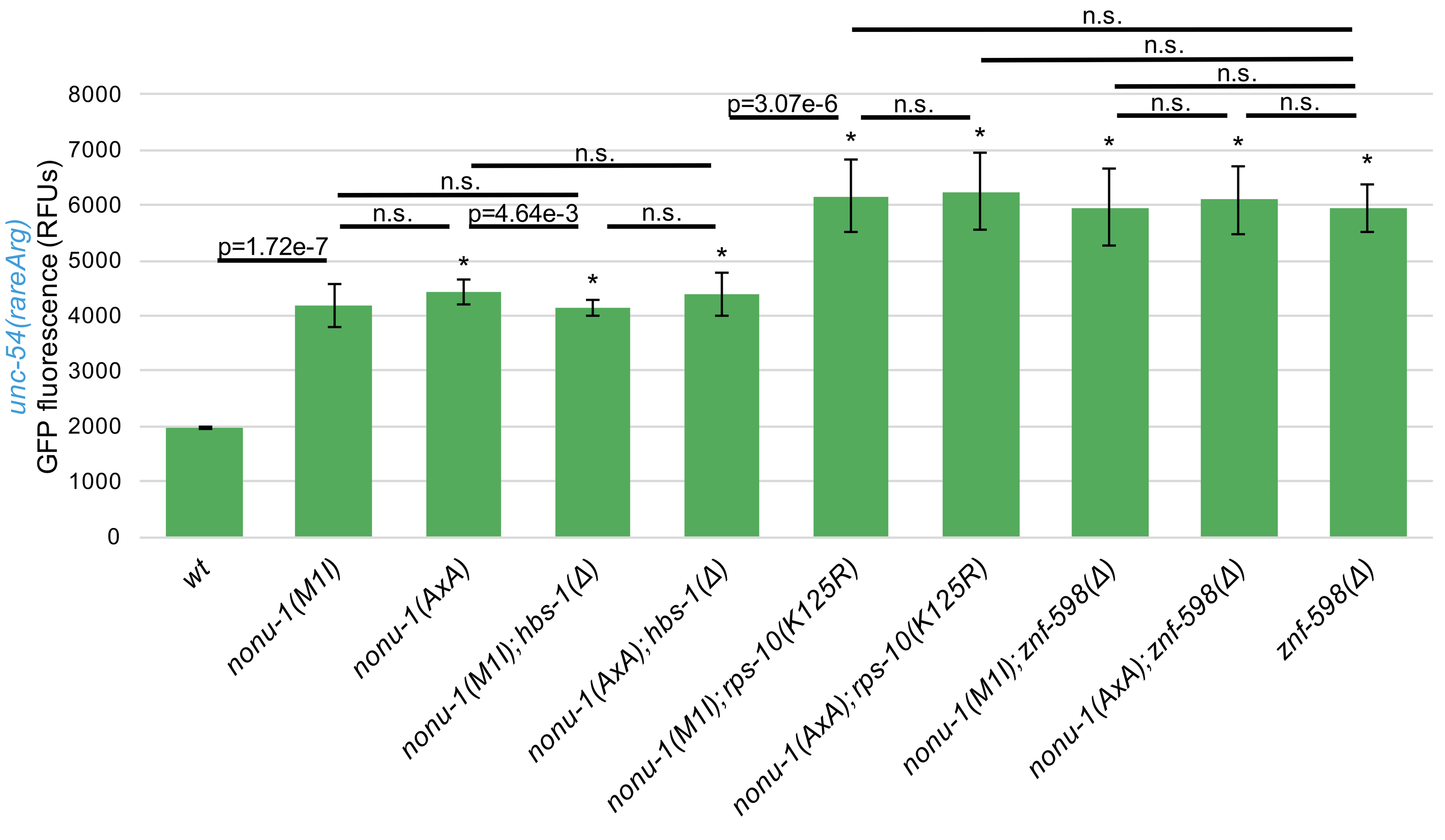

Supplement: S3 Fig — Mean RFUs (relative fluorescence units) of indicated strains (n≥15 animals/strain) in the unc-54(rareArg) background. One standard deviation shown as error bars. p values from Welch’s t-test, with asterisks indicating p<0.01 for all comparisons with wild type. (TIF) [file pgen.1010577.s005.tif]

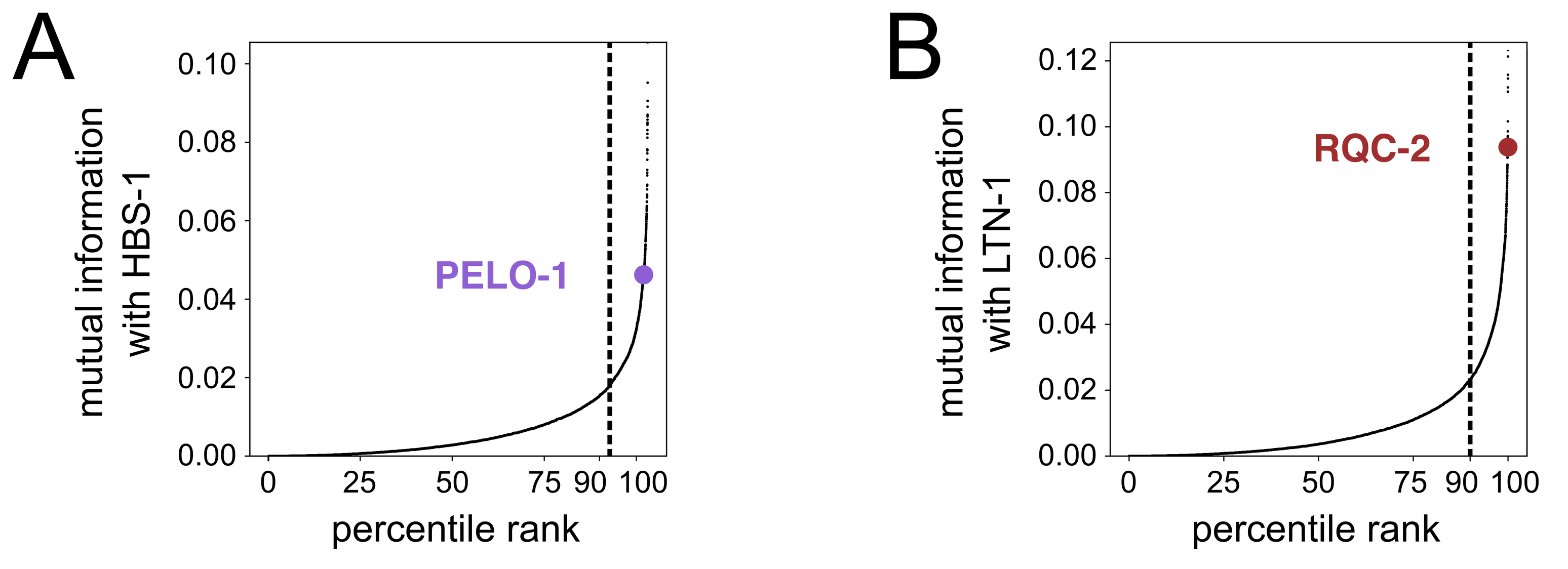

Supplement: S4 Fig — (A) HBS-1 mutual information plot. 90% percentile cutoff is shown as a dashed line and PELO-1 is highlighted in purple. (B) As in (A), showing LTN-1 mutual information with RQC-2 in red. (TIF) [file pgen.1010577.s006.tif]

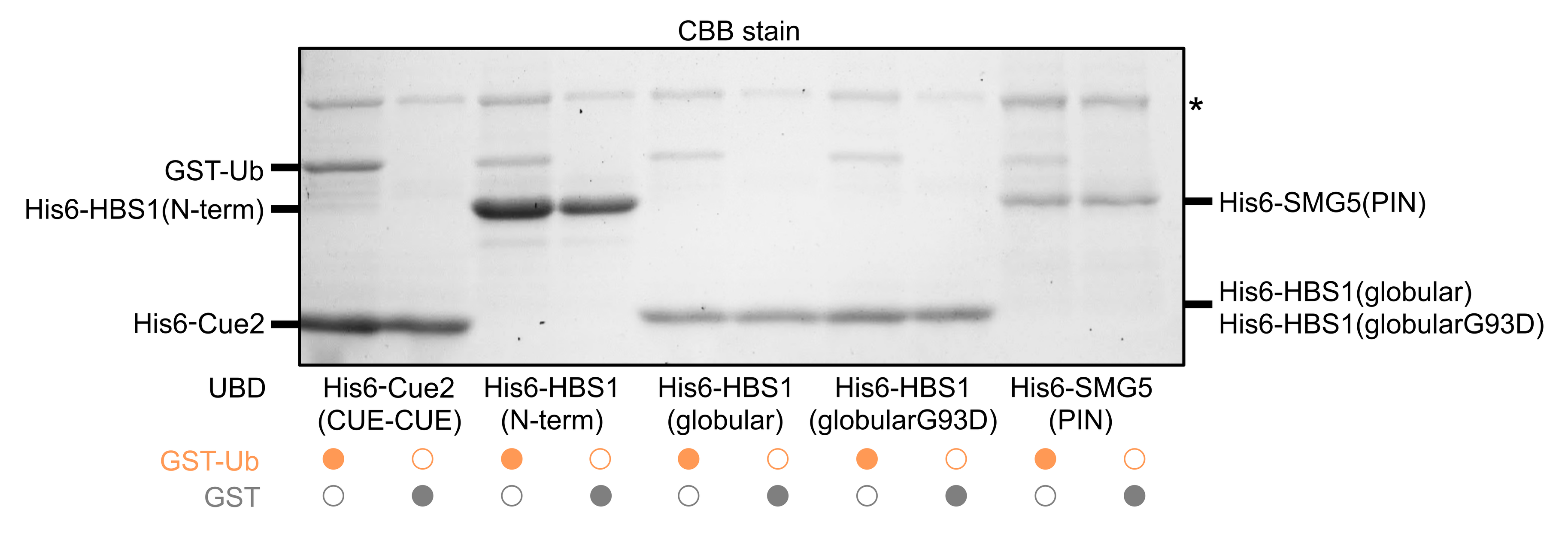

Supplement: S5 Fig — His6-tagged constructs of S. cerevisiae Cue2 CUE domains, H. sapiens HBS1 N-term domain, H. sapiens HBS1 N-term globular (triple helix) domain, H. sapiens HBS1 N-term globular (triple helix) domain with G93D mutation (Gly in predicted binding site found from [55]), and H. sapiens SMG5 PIN domain. His6 constructs were immobilized on cobalt metal affinity resin and incubated with E. coli lysates expressing GST-Ub or GST. Proteins boiled from resin are shown on Coomassie stained gel, with proteins indicated. Asterisk indicates nonspecific peptide present on resin. (TIF) [file pgen.1010577.s007.tif]

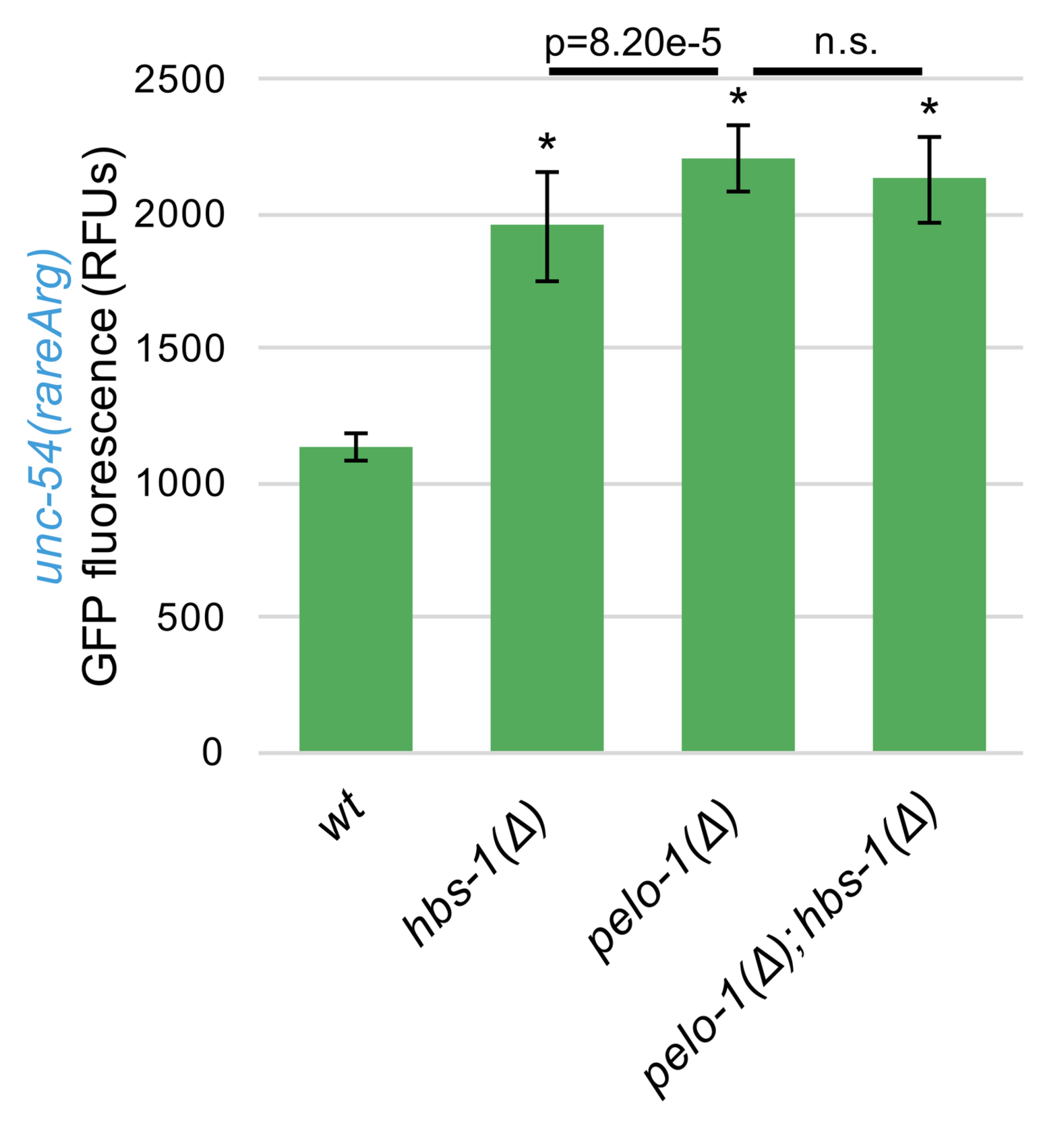

Supplement: S6 Fig — Mean RFUs (relative fluorescence units) of indicated strains (n≥15 animals/strain) in the unc-54(rareArg) background. One standard deviation shown as error bars. p values from Welch’s t-test, with asterisks indicating p<0.01 for all comparisons with wild type. (TIF) [file pgen.1010577.s008.tif]
