# Supplementary material for: Ubiquitination of stalled ribosomes enables mRNA decay via HBS-1 and NONU-1 in vivo
Source: PLoS Genet. 2023 Jan 10;19(1):e1010577. doi: 10.1371/journal.pgen.1010577 (PMC9870110; doi:10.1371/journal.pgen.1010577)
Supplement: Attachment — Submitted filename: 221129_responseToReviewers.pdf [file pgen.1010577.s009.pdf]

Reviewer #1: In this manuscript the authors study no-go and nonstop mRNA decay in C. elegans. They use the powerful genetics of this system to identify factors required for the two pathways and then follow up with experiments that provide strong evidence that supports the main conclusion that "Ribosomal ubiquitination facilitates mRNA cleavage and ribosome rescue during No- Go and Nonstop mRNA Decay". Unfortunately, the same conclusion has previously been reached for yeast and other metazoan systems, and thus this main conclusion was to be expected. The main contribution is to extend these observations to C. elegans (and for the first time an intact animal). The novel aspect of this manuscript is that the authors speculate that ribosome ubiquitination recruits Hbs1 through an N-terminal ubiquitin binding domain. This novel part is very preliminary and only based on sequence gazing. Finally, the authors report some ribosome profiling that do not lead to clear conclusions and thereby weaken the overall manuscript.

We thank the reviewer for their critical feedback. In response, we have expanded the manuscript's genetic findings pertaining to NGD, and been more explicit as to how our work expands from what was known. We have added a more thorough analysis of HBS-1 and its place in NGD, expanding the scope of the work. We removed the SKI work and the ribosome profiling as we feel that it is more adequately dealt with in its own right (and in response to feedback that it weakens the manuscript). Below we describe the changes point-by-point. We hope the reviewers will agree that the end product is substantially improved and significantly adds to the literature.

Major comments:

1. While the genetic screens form the basis for the paper and one of its strengths, their description is brief and incomplete. The screen selects for suppressors of the unc-54(srf1004) allele, but the exact sequence of this allele is not given, and no detail are provided on how it was created. The methods describe two screens with the initial screen only identifying znf-598 mutants. This is not explained in the text. Is this simply because the nonu-1 loss has a smaller effect size? The text describes that for the mutants "In each case we observed de-repression of the reporter, manifest as increased fluorescence and an improvement in animal movement and egg laying." This is key data for the paper and should be shown.

Thank you for this suggestion. We have included a more detailed description of the *unc-54(rareArg)* screen in the main text, including an explanation that the non-*znf-598* mutants had less pronounced phenotypes. See paragraph starting with "Using *unc-54(rareArg),* we performed two genetic screens…"

2.The authors mutate Lys residues in rps-10 and rps-20 to Arg and ascribe any effects on the absence of ubiquitination. The authors do not show that these mutant rps proteins are expressed at their normal level and that the proteins fail to be ubiquitinated. Simply adding a rps10::HA(K125R) lane to figure 3B would greatly strengthen the data, as would addition of a similar blot for rps-20. The authors should add whether these mutations

cause any organismal phenotype that is different from znf-598 to address whether the point mutant ribosomes are otherwise functional.

We agree that it is important to show expression of our mutant ribosomal proteins. We have included these key data, and shown that ribosomal protein expression is not grossly perturbed by the lysine>arginine substitutions. We incorporated these data into the revised manuscript (Fig 3C), updated the text, including commenting on the fact that these animals are healthy. Our original interpretation of the role of ribosomal ubiquitination remains unchanged and has indeed been strengthened by this suggestion.

3. The manuscript uses a catalytic mutation in nonu-1 [nonu-1(AxA)] for several key experiments. Supposedly this protein would still be able to bind stalled ribosomes, and might be dominant negative for some aspects. How do the effects of the nonu-1(AxA) allele compare to a nonu-1 null allele?

We shared this concern. We repeated several of our key findings with a *nonu-1* null allele (M1I) and observed identical effects to the catalytic (AxA) mutant (Fig S3). Our original conclusions remain unchanged.

4. The authors argue on page 24 that based on the number of alleles of skih-2 and ttc-37 they isolated in the nonstop screen, they should have isolated alleles of a gene that encodes Ski7 function if it exists. It is noteworthy that in yeast Ridley et al 1984 (PMID: 6371496) isolated 25 ski2 alleles and 22 ski3 alleles, but only 2 ski7 alleles. Similarly, there are dozens of human trichohepatoenteric syndrome patients with mutations in SKIV2L and TTC37, but none with mutations in HBS1LV3.

Thank you for this feedback. We have removed the SKI complex figures from this manuscript.

5. Some of the figures indicate statistical significance, but this is not consistently shown. For example in Figure 3H, the authors indicate that the znf-598 data are significantly different from wt. I assume the same is true for nonu-1(AxA) but that is not indicated. More importantly, is the znf-598 nonu-1 double mutant different from either single? The authors imply in the text that they are ("The fact that nonu-1 and znf-598 together exhibit additive effects").

Thank you for this suggestion. We annotated statistical significance throughout the figures (see updated Fig 3B, Fig 4A, Fig 5B, Fig 5C, Fig 5G, Fig 6D, Fig 7B, Fig 7C, Fig 7F, Fig S3, Fig S6).

6. I could not find how many biological replicates were performed for most of the experiments.

Thank you for the opportunity to clarify; we added n values in the figure legends, and briefly describe them here: for overexpression experiments, we used 3 independent genetic isolates, with at least 4 images of each isolate; for GFP fluorescence experiments, we imaged at least 15

independent animals of the same genotype; for RNA-seq experiments, we performed two biological replicates.

7. In the RNA-seq data of figure 3F, unc-54(rareArg) is clearly affected, but it appears that very few if any other genes are. Can the authors comment on this? Does this mean znf-598 does not have any endogenous targets, or that the 12 Rare Arg residues cause stalling beyond the normal physiological range, or something else?

The short answer is that very few endogenous genes change to the extent of *unc-54(rareArg)*. As the reviewer keenly points out, this could be for several reasons, but it will take some time for us to unpack and distinguish between these possibilities. We look forward to elaborating on this ("the long answer") in a separate manuscript (Monem et al., *in prep*).

8. The section "Structural, computational, and functional evidence for ubiquitin-binding by HBS-1" is highly speculative and despite the section heading does not contain any "functional evidence" and this section should probably be deleted. Alternatively, it requires substantial experimental evidence to show that the hbs-1 N-terminus indeed binds ubiquitin or ubiquitinated proteins.

Our updated manuscript expands on and clarifies the function of the HBS-1 N-terminus. Despite the HBS-1 N-terminal domain's homology to ubiquitin-binding domains (Fig 6A, Fig 6B, Fig 6C), we observed no binding to ubiquitin *in vitro* (Fig S5). Moreover, we observed that the HBS-1 N-terminus is dispensable for repression from ribosomal stalling (Fig 6D). We've included these findings and updated our manuscript accordingly (see paragraph starting with "To determine whether the N-terminus of HBS-1 is required…" and "While our genetic analyses support a role…").

9. It is not clear that the last two sections of the results "Deletion of ZNF-598 increases mRNA levels of and ribosomes on a No-Go mRNA Decay substrate" and "Ribo-seq libraries lack accumulations at arginine stalls" add to the manuscript. The main conclusion the authors seem to draw is that "standard monosome and disome Ribo-seq protocols are unable to capture metazoan ribosomes at strong stalls." If the main conclusion is indeed that the experiments in figure 5 and 6 are uninformative for technical reasons they probably shouldn't be a main focus of the main results section. I do understand that there is some value to publishing this information, but by appending it to this paper it distracts from the main point.

We have removed this section from this current manuscript.

10. Table S1: C. elegans strains and oligos is missing from the version I was provided.

Please see the attached Table S1 in this version.

Minor comments:

11. The authors should pay closer attention to referencing. examples include:
page 3 “the field has recently hypothesized…” should have references
page 9 “REF Halbach et al. 2013 23953113” should be “Halbach et al. 2013” (similar issues on page 11)
page 11 “Ski7 function may be encoded via alternative splicing of the hbs-1 gene as it is in several organisms” should have references.

Thank you for the keen eyes. Removed from the manuscript.

12. Can the authors comment on why they only identify missense mutations in skih-2 in their nonstop screen, and only nonsense mutations in its partner ttc37?

Removed from the manuscript.

13. Figure 5 shows 5 ttc-37 alleles. The text (pages 5 and 7) indicate only 4 were isolated.

Removed from the manuscript.

14. “cerevisiae” and “sapiens” should be spelled out in the text instead of S. cer. and H. sap.

Done.

15 The authors describe C89 of znf-598 as “catalytic”, but as far as I know this residues is structural, not catalytic.

Thank you–changed.

16. The results section “Exosome divergence at the Ski7 interface in C. elegans” and figure 2 does not contain any results and at a minimum should be moved to the discussion or supplemental materials. This section only relates to the nonstop mRNA screen and thus is not connected to the main topic of the manuscript.

Removed from the manuscript.

17. Starting on page 15 and continuing on p17 the authors mention a Gly residue in S. cerevisiae Hbs1. Missing is which Gly of S. cerevisiae Hbs1 they refer to and whether this residue is conserved. Is there any evidence that this Gly is required for yeast Hbs1 function?

Removed from the manuscript.

18. On page 17 "In both structures, the HBS-1 N-terminus is bound to the 40S subunit near uS10 and uS3 (Hilal et al., 2016; Becker et al., 2011)" is offered as support for the idea that the Hbs1 N-terminus binds ubiquitinated ribosomes, but the data in the two references appear to support that this domain binds un-ubiquitinated ribosomes. Is there any density in the structural data that suggests ubiquitination? Have the authors entertained the idea that rps-10 or rps-20 ubiquitination could block binding of the Hbs1 N-terminus instead?

Thank you for this insightful comment. We have considered this idea in the Discussion (see paragraph starting with "Here, we discovered that the N-terminal domain of HBS-1 is dispensable for NGD…".

19. on page17 the authors wrote "We also noticed that plant HBS-1 homologs contain a conserved C4-type zinc finger homologous to a known Ub-binding domain from rat (Fig 4C) (Alam et al., 2004). Thus, we hypothesize that the Ub-binding function of HBS-1 emerged relatively early in the evolution of this factor." It is not clear to me how the addition of a C4-type zinc finger to plant Hbs1 leads the authors to hypothesize that the Ub-binding function of HBS-1 emerged early. As far as I can tell the C4-type zinc finger shows that is the focus of figure 4C shows no similarity to the CUE/Aha/Hbs1-N domain of figure 4A and B and it is not at all clear how this clarifies function of Hbs1

Thanks for the opportunity to clarify our language and our thinking. Indeed, the result supports no hypothesis on the timing of the evolution of this domain. "These observations show that HBS-1 N-termini from diverse organisms lack sequence or structural homology with each other, and yet many N-termini instead share homology with domains that bind ubiquitin." We have added this sentence to the results section (see paragraph starting with "The HBS-1 protein consists of an N-terminal domain…").

Reviewer #2: In the manuscript, Monem et al. utilized their own Nonstop mRNA Decay reporter worms to demonstrate that SKI complex subunits SKIH-2 and TTC-37 are required for efficiently repressing translation of the Nonstop mRNA. The authors also generated a novel No-Go mRNA Decay reporter worms that rely on a rare Arg codon stretch and confirmed that an E3 ubiquitin ligase ZNF-598 and an endonuclease NONU-1, known factors for No-Go mRNA Decay, are required for efficient repression of the reporter. Genetic screening with the No-Go mRNA Decay reporter further identified uba-1 encoding the sole E1 ubiquitin-activating enzyme in C. elegans. hbs-1 encoding a GTPase homologous to eRF3 was identified in both the Nonstop mRNA Decay screen and the No-Go mRNA Decay screen. PELO-1/Dom34 (homologous to eRF1) was also required for efficient repression of both the reporters. Cys89 of ZNF-598 was crucial for ubiquitination of RPS-10, an rps-10 (K125R); rps-20 (K6R+K9R) double mutant phenocopied the znf-598 (null) mutant in de-repression of the No-Go mRNA Decay reporter and an rps-10 (K125R) znf-598 (null) double mutant was comparable to the

znf-598 (null) single mutant, indicating that ubiquitination of these ribosomal proteins by ZNF-598 is critical for No-Go mRNA Decay. A catalytic mutation in nonu-1 did not enhance de-repression of the znf-598 (null) mutant implying that they function in the same pathway. hbs-1 (null) mutation did not enhance de-repression of the znf-598 (null) mutant either. RNA-seq and Ribo-seq analysis revealed that the No-Go mRNA Decay reporter mRNA was stabilized in the znf-598 (null) mutant. However, the distributions of monosomes and disomes were not affected upon de-repression from No-Go mRNA Decay.

1. The Nonstop and No-Go mRNA Decay reporters are unique and excellent for studying mRNA surveillance system in vivo and enabled the authors to characterize the factors required for efficient repression of the problematic mRNAs. It seems to me, however, that the manuscript relies too much on assumption that molecular mechanisms of No-Go mRNA Decay in C. elegans is similar to those in other species such as yeast and human, leading to biased interpretation of the results. The readers would be interested in whether or not this independent genetic study with the excellent reporter system in C. elegans supports the model from other species.

Thank you for the opportunity to clarify. We think there are similarities as well as differences. In *C. elegans*, our analysis of ZNF-598-dependent ubiquitination sites on ribosomal proteins suggest that *C. elegans* detects and responds to stalling in a manner similar to human cells. See paragraph starting with “In the NGD screen, we recovered several alleles of *znf-598*…”. In *C. elegans*, we can clearly see strong phenotypic effects with knockout of *nonu-1*, whereas in yeast knockout of the homologous *CUE2* does not have effects (unless other factors are also knocked out). This is a difference that is not yet understood, and given the lack of studies on the human homolog (N4BP2), we do not know whether humans are similar or different from either organism. See paragraph starting with “To initially validate the *unc-54(rareArg)* reporter…”.

2. In Abstract, the authors claim that “we present data consistent with a model where ubiquitination recruits the endonuclease NONU-1 via CUE domains and the ribosome rescue factor HBS-1 via its poorly characterized N-terminus” (lines 27-29). The provided results are actually consistent with the model, but do not preclude other models. Biochemical analysis and/or genome editing to disrupt critical residues are necessary to claim the model. “Our work (1) presents a model for how ribosomal ubiquitination directly causes mRNA decay and ribosome rescue” (lines 29-30). The evidence for this claim is also weak (see below). It is still unclear whether ribosomes are actually rescued by HBS-1 upon No-Go mRNA Decay in C. elegans.

Thank you for this concern. In the updated manuscript, we added genetic analysis of mutants to test ubiquitin-binding by both NONU-1 (deletion of the CUE domains) and HBS-1 (deletion of the N-terminal domain). Our additional data supports ubiquitin-binding by NONU-1 (Fig 5B), and are consistent with our previous conclusions. As for ubiquitin-binding by HBS-1, we observed the N-terminal domain to be dispensable for NGD in our system (Fig 6D), and its ubiquitin

binding was comparable to background *in vitro* (Fig S5). For more on HBS-1, please see our response to Reviewer #1 point 8.

3. The genetic evidence provided in the manuscript do not determine the order of genes in the Model for No-Go mRNA Decay shown in Figure 4H. Gain of function or overexpression of the upstream genes should be suppressed by loss of function of the downstream genes. Gain of function or overexpression of the downstream genes should suppress loss of function of the upstream genes.

Thank you for this great suggestion. The revised manuscript considerably expands on this area. We have performed several additional genetic experiments to test the model, specifically, overexpression as the reviewer suggested (Fig 2A, Fig 2B, Fig 5D, Fig 5E, Fig 7D, Fig 7E). Our genetic analyses of *nonu-1* places it downstream of *znf-598* (Fig 5D, Fig 5E). Therefore, our previous conclusions remain unchanged (see paragraph starting with “We next investigated the ordering of ZNF-598 and NONU-1 relative to one another…”). Our genetic analyses of *hbs-1* revealed a role for this factor downstream of *znf-598* (Fig 7D) and upstream of *nonu-1* (Fig 7E). Our RNA-seq analyses of *hbs-1* and other mutants substantiate a role for HBS-1/PELO-1 in facilitating the RNA cleavage reaction brought about by NONU-1/Cue2. Our updated manuscript contains these findings, as described in the results and discussion (see paragraphs starting with “HBS-1 and PELO-1 are predominantly known…” and “Given prior work in *S. cerevisiae* revealing a role for Hbs1 in NGD cleavage…”). This significantly expands the scope and novelty of our findings.

Specific points:

1. Line 140, “(Fig S1)”: Please specify as “Fig S1A”.

Removed.

2. Line 142, “The majority of the skih-2 suppressors were in the catalytic RecA domains of this protein, or lined the mRNA binding channel (Fig S1).”: Please specify as “Fig S1B”. It is not clear in Figure S1B where these domains are.

Removed.

3. Line 148, “no stop codons (burgundy)”: I cannot see the burgundy region.

Removed.

4. Line 155, “(E) znf-598, nonu-1, and uba-1 alleles drawn as in part (B).”: The order of the genes should be as in the Figure.

Done.

5. Lines 163-164, “All four of the novel ttc-37 suppressors were premature stop codons”: Please cite Figure 1B. Five alleles are drawn in the Figure. Please clarify what “SS” indicates.

Removed.

6. Lines 167-168, “We observed a similar fold de-repression in ttc-37 and skih-2 mutants (Fig 1C).”: It is not explained why ttc-37 (SS) and skih-2 (delta) were used in this experiments. Because all the skih-2 mutants have missense alleles, it should be clarified to what extent such missense mutations affect expression of the reporter. Fluorescence microphotographs of the mutant worms would help.

Removed.

7. Lines 168-169, “we observed epistasis: a skih-2 ttc-37 double mutant exhibited a similar fold de-repression to the single mutants”: The term epistasis is not used here in a classical way.

Removed.

8. Lines 187-188, “we crossed it with alleles of two factors known to be required for No-Go mRNA Decay: znf-598 and nonu-1”: Please specify the allele used in the experiment.

Done.

9. Line 194 and many others, “S. cer”: Please spell in full.

Done.

10. Line 206, “highly conserved GHP motif”: A bit more information about the motif.

Removed.

11. Lines 219-220, “Requirement of hbs-1 in both pathways was confirmed by crossing in a deletion allele constructed by CRISPR/Cas9”: It would be nice to see GFP expression in supplemental figures.

Thank you–a fluorescence image of an *hbs-1* mutant in *unc-54(rareArg)* is included (Fig 1C) and GFP quantification is included (Fig 7B, Fig 7C).

12. Lines 220-221, “GTP binding and active site residues”: It would be nice to see amino acid sequence alignment.

Thank you for your suggestion. We have included a multiple sequence alignment of *C. elegans* G200 (identified in our screen) along with the yeast and human homologs (Fig 1C).

13. Lines 224-225, “In hbs-1 and pelo-1 mutants, we observed consistent de-repression of unc-54(nonstop), and a similar pattern in unc-54(rareArg).”: Please specify the alleles. Please show or cite the data.

Thank you–*unc-54(rareArg)* data is included in Fig S6.

14. Line 226, “Interestingly, in both reporters the effect of hbs-1 was slightly less than the effect of pelo-1”: Figure 1G should be cited.

Removed.

15. Line 237 and others, “REF Halbach et al. 2013 23953113”: Incompletely formatted.

Removed.

16. Lines 260-261, “periods indicate amino acids with weakly similar properties”: No periods in the Figure.

Removed.

17. Lines 266-267, “our hbs-1 deletion exhibited a phenotype similar to pelo-1 mutations, not skih-2 nor ttc-37 mutations”: Please cite Figures 1C and 1G.

Removed.

18. Lines 328-329, “Our genetic results support a model where NONU-1 functions downstream of ZNF-598 in No-Go mRNA Decay,”: Please describe the results in the text.

Thank you–done. Please see paragraphs starting with “To determine whether NONU-1 acts in the same pathway as ZNF-598…” and “We next investigated the ordering of ZNF-598 and NONU-1 relative to one another…”.

19. Line 332, “we observed an increase of the unc-54(rareArg) reporter mRNA in znf-598”: Please specify how much fold the mRNA is increased.

Done. Please see Fig 7A.

20. Line 362, “double mutant expressed higher levels of GFP”: Please cite Figure 3H.

Done.

21. Line 367, “Several of our No-Go mRNA Decay suppressors (znf-598, nonu-1, uba-1) encoded factors”: The term “suppressor” is used in inconsistent ways, sometimes as a mutation that suppress No-Go mRNA Decay, sometimes as a gene whose mutations can suppress No-Go mRNA Decay.

Done. We have used clearer language when discussing the mutations and genes identified in our screens.

22. Line 433, “Our genetic analyses substantiated the notion that HBS-1 functions through ubiquitin binding.”: Biochemical evidence is desirable.

Thank you for this suggestion. We performed experiments to further test this hypothesis. We observed the N-terminal domain to be dispensable for NGD in our system (Fig 6D), and we observed no binding to ubiquitin *in vitro* (Fig S5). For more on HBS-1, please see our response to Reviewer #1 point 8.

23. Line 435, “Taken together, our structural, computational, and genetic observations support the idea that ubiquitination is critical for ribosome rescue by HBS-1 (Fig 4H).”: No evidence was presented for ribosome rescue by HBS-1. The order of genes in the model should be validated by genetic studies with gain of function mutants.

Thank you for this concern. The manuscript has been significantly revised to incorporate additional genetic experiments to further test this model. Namely, we included overexpression experiments to delineate the ordering of genes (Fig 2A, Fig 2B, Fig 5D, Fig 5E, Fig 7D, Fig 7E). Please see our response to the earlier main point #3.

24. Lines 440-441, “Given our evidence that znf-598 functions upstream of both mRNA decay (via NONU-1) and ribosome clearance (via HBS-1),”: Same as above.

Thank you–please see our response to the above main point #3.

25. Line 448, “monosome footprint count is a reasonable predictor of the abundance in the captured disomes”: Not clear whether this result was expected.

Removed.

26. Figure S1A, lines 18-19, “Breaks in the alignment 18 are indicated with ellipses.”: Not clear what “ellipses” indicate. It is not explained what asterisks, colons and periods indicate.

Removed.

27. Figure S2B, lines 27-28, “p values from Student's t-test.”: p values are not indicated in the figure.

Done–we have annotated statistical significance throughout the figures (see updated Fig 3B, Fig 4A, Fig 5B, Fig 5C, Fig 5G, Fig 6D, Fig 7B, Fig 7C, Fig 7F, Fig S3, Fig S6).

28. Figure S9, lines 77-79, "(A) Scatter plots showing read coverage of 10nt sequences mapping to the wildtype unc-54 ORF from RNA-seq, monosome Ribo-seq, and disome Ribo-seq libraries.": Should be mentioned as appear in the Figure.

Thank you for noticing this, it has been removed.

Reviewer #3: In this manuscript entitled "Ribosomal ubiquitination facilitates mRNA cleavage and ribosome rescue during No-Go and Nonstop mRNA Decay", Monem, Arribere and colleagues reported functions and mechanisms of key factors of nonstop (NSD) and no-go (NGD) mRNA decay. They first identified novel and known NSD and NGD factors by performing mutagenesis screens using reporter systems. They showed that ubiquitination of collided ribosomes by ZNF-598 was required for the clearance of stalled ribosomes and for NGD. They also demonstrated that key functionally paired factors of NSD and NGD, including HBS-1 and PELO-1, were broadly conserved across species. By performing ribo-seq analysis, the authors found that ZNF-598 and HBS-1 were required for NGD, and standard ribo-seq methods were unable to capture stalled ribosomes at strong stall-inducing sequences. This elegant and rigorous paper provides crucial information for the mechanism by which NSD and NGD factors degrade target RNAs. Following are my remaining minor comments to help improve this excellent paper.

1. I suggest that the authors briefly explain NMD in the introduction, in particular because they performed experiments with smg-1 for figure S7B.

Thank you for this suggestion. Mentions to NMD have been removed from the manuscript.

2. The genetic interaction experiments are very nice and the results are mostly clear. However, as they tested genetic interaction between loss-of-function mutants for NSD and/or NGD. Thus, I don't think they can say line 168: "we observed epistasis", and please change the wording.

Thank you–we have changed our wording.

3. Related to my comment 2, is there any potential gain-of-function mutant for NSD or NGD? Can they add Discussion point about this?

Thank you for this concern. We have constructed an overexpression system to address this, and substantially expanded the manuscript. Please see our response to Reviewer #2's main point #3.

4. I like their mutual information analysis. However, all their analysis produces high mutual information. I wonder if they can show some "negative controls", which do not produce high mutual information? That will make their positive results more specific and valid.

We shared this concern. We have included negative controls on the mutual information plots. Please see Fig 5H, Fig 7H, Fig 7I.

5. I found several small errors in this manuscript. Following are some examples, and I recommend that the authors carefully proofread the manuscript for correcting typos and errors.
1) On page 3, line 73, please add "+ " between "AAA" "ATPase".

Thank you–removed.

2) On page 33, line 812, please doublecheck whether 1.0x is correct.

Thank you, 1.0x on the AxioZoom is correct.

3) On page 35, line 872, please correct the concentration of 1x SDS loading buffer, not the stock material concentration.

Thanks–done.

4) Please add ";" between genes of different chromosome when the authors write double mutants (for example, skih-2; ttc-37).

Removed.

5) Please use full names instead of abbreviations for the main text (e.g. C. elegans rather than C. ele).

Done.

6. I recommend the authors doublecheck citations in this manuscript. Following are some examples.
1) On page 7, line 183, "personal communication"
2) On page 9, line 237, "REF Halbach et al. 2013 23953113"
3) On page 11, line 273 "REF Kowalinski et al., 2016 27345150"
4) In Figure 4 legend, "Burschowsky et al., unpublished"

Thanks for noticing these, some are removed, others revised.

7. It will be nice if the authors add more details to the proposed model in Figure 4H, and may want to make a separate last figure 7.

Thank you for this suggestion. We have revised our model figure. Please see Fig 7G.
