# Supplementary material for: Ubiquitination of stalled ribosomes enables mRNA decay via HBS-1 and NONU-1 in vivo
Source: PLoS Genet. 2023 Jan 10;19(1):e1010577. doi: 10.1371/journal.pgen.1010577 (PMC9870110; doi:10.1371/journal.pgen.1010577)
Supplement: Attachment — Submitted filename: 221214_responseToReviewers.docx [file pgen.1010577.s010.docx]

**Reviewer #1: The authors successfully addressed my remaining concerns and the manuscript has been greatly improved.**

Thank you for the response.

**Reviewer #2: This is a very thoroughly revised manuscript that is greatly improved by the addition of new data, removal of data irrelevant to the main point, and by thoroughly revising the text. In this manuscript the authors study no-go mRNA decay in C. elegans. They very effectively use the powerful genetics of this system to identify factors required for no-go decay and then follow up with experiments that provide strong evidence that supports the main conclusion that ribosomal ubiquitination enables mRNA degradation via HBS-1 and NONU-1 in an intact animal. The revised manuscript is a valuable extension of what is known and should be published in PLOS Genetics. I have some very minor comments for the authors that may help them further improve the manuscript detailed below.**

**Minor comments:**

**1. The authors isolated 20 mutants but only described 9 alleles. If the authors identified mutations in the other 11 mutants, it would be helpful to the field to add those to table S1, for example if another researcher identifies mutations in the same genes or their orthologs. If the other 11 alleles were not mapped and sequenced, perhaps the authors can clarify that.**

We have included in Table S1 five additional screen mutants which will be more thoroughly described elsewhere. The remaining six screen mutants have yet to be mapped. Please see the appended sentence: “Five alleles in one additional gene…”.

**2. In line 298 the authors mention creating tagged alleles of NONU-1 that turned out to be nonfunctional. It might be useful to the field to include the details in the supplement. This would prevent future researchers from wasting time trying the same tagging strategy.**

We have included these allele sequences in Table S1 and further clarified the tags in the main text. Please see the revised sentence: “We also attempted to examine expression of NONU-1 by tagging the N-terminus (Table S1)...”.

**3. The use of “phenocopy” in lines 223, 285, 307, 308, and 427 is at best unnecessary jargon but according to the definition of phenocopy in my dictionary or in Wikipedia is actually incorrect. Phenocopy should mean that some specific treatment (e.g. a drug) has the same effect as a mutation and not that two mutations have the same effect. The authors correctly describe mutations with similar phenotypes in lines 296 and 403-406. Even worse is in line 389 where a mutant is described as phenocopying wild type. Much clearer would be to say that deleting the N-terminus of HBS-1 had no discernible phenotype.**

Thank you for helping us clarify these sections. Please see the following revised sentences: “The *znf-598(C89A)* mutant displayed *unc-54(rareArg)* de-repression indistinguishable from the phenotype of *znf-598(Δ)*...”, “The fact that loss of *znf-598* can be mimicked by mutation of ribosomal proteins...”, “We combined mutations in *znf-598* and ribosomal ubiquitination sites with *nonu-1(AxA)*...”, “The *nonu-1*; *hbs-1* double mutant was indistinguishable from a *nonu-1* single mutant…”, and “Surprisingly, when measuring unc-54(rareArg) GFP levels, deleting the N-terminus of HBS-1 had no discernible phenotype…”.

**4. Line 189 “containing fluorescent protein fusions” is incorrect. The T2A stop and go sequence means that mCherry and ZNF-598 (or NONU-1) are produced as separate proteins and not fusion proteins. In addition, plasmids may encode proteins, but do not “contain” them.**

Thank you for noticing this wording. Please see the revised sentence: “We constructed plasmids encoding fluorescent proteins…”.

**5. Line 429 “acts” should be “act”**

Thank you for finding this typo. Fixed.
